# Piggybacking on Classical Import and Other Non-Classical Mechanisms of Nuclear Import Appear Highly Prevalent within the Human Proteome

**DOI:** 10.3390/biology9080188

**Published:** 2020-07-23

**Authors:** Tanner M. Tessier, Katelyn M. MacNeil, Joe S. Mymryk

**Affiliations:** 1 Department of Microbiology and Immunology, The University of Western Ontario, London, ON N6A 3K7, Canada; ttessie2@uwo.ca (T.M.T.); kmacne9@uwo.ca (K.M.M.); 2 Department of Otolaryngology, Head & Neck Surgery, The University of Western Ontario, London, ON N6A 3K7, Canada; 3 Department of Oncology, The University of Western Ontario, London, ON N6A 3K7, Canada; 4 London Regional Cancer Program, Lawson Health Research Institute, London, ON N6A 5W9, Canada

**Keywords:** nucleus, import, export, NLS, importin, piggyback, co-transport, mediator, TFIID

## Abstract

One of the most conserved cellular pathways among eukaryotes is the extensively studied classical protein nuclear import pathway mediated by importin-α. Classical nuclear localization signals (cNLSs) are recognized by importin-α and are highly predictable due to their abundance of basic amino acids. However, various studies in model organisms have repeatedly demonstrated that only a fraction of nuclear proteins contain identifiable cNLSs, including those that directly interact with importin-α. Using data from the Human Protein Atlas and the Human Reference Interactome, and proteomic data from BioID/protein-proximity labeling studies using multiple human importin-α proteins, we determine that nearly 50% of the human nuclear proteome does not have a predictable cNLS. Surprisingly, between 25% and 50% of previously identified human importin-α cargoes do not have predictable cNLS. Analysis of importin-α cargo without a cNLS identified an alternative basic rich motif that does not resemble a cNLS. Furthermore, several previously suspected piggybacking proteins were identified, such as those belonging to the RNA polymerase II and transcription factor II D complexes. Additionally, many components of the mediator complex interact with at least one importin-α, yet do not have a predictable cNLS, suggesting that many of the subunits may enter the nucleus through an importin-α-dependent piggybacking mechanism.

## 1. Introduction

The nucleocytoplasmic transport of proteins across the nuclear envelope is an essential cellular process unique to eukaryotic organisms. The nuclear envelope spatially separates the contents of the nucleus from the cytoplasm, and provides a physical mechanism for regulating numerous cellular events such as transcription, translation and the cell cycle. The transport of proteins and RNA across the nuclear envelope is a tightly orchestrated process that requires all molecules to pass through the nuclear pore complex (NPC), a large multimeric complex built from multiple copies of approximately 30 different proteins called nucleoporins [1,2]. In essence, the NPC functions as a semi-permeable barrier, selectively allowing the passage of certain molecules while simultaneously preventing the passage of others [3]. Proteins of varying sizes are able to diffuse through the central channel of the NPC; however, this is influenced by several factors. Notably, the rate at which a protein can diffuse into the nucleus is inversely related to its size. As protein size increases from <30 to 40 kDa, its ability to diffuse diminishes rapidly [4,5]. Other factors, such as a protein’s shape and surface composition, also have a significant effect on passive diffusion [4,6]. Nevertheless, despite proteins having the capacity to diffuse through the NPC, proteins of all sizes employ the assistance of nuclear transport receptors for rapid and efficient nuclear import or export [7,8].

The bidirectional transport of proteins through the NPC is carried out by a group of soluble transport receptor proteins belonging to the karyopherin-β (Kapβ) superfamily, which can be further subdivided into importins or exportins [9]. The human genome encodes 20 Kapβ proteins; 10 are importins that shuttle proteins into the nucleus, 7 are exportins and shuttle proteins out of the nucleus, 2 are bidirectional transporters, and 1 currently has no known function [10]. Kapβ proteins recognize a cargo’s nuclear localization signal (NLS) for import, or its nuclear export signal (NES) for proteins which undergo export [11,12]. Following cargo binding, Kapβ can facilitate transport through the NPC, where cargo is released into either the cytoplasm or nucleus. Importantly, the loading and un-loading of cargo onto Kapβ is aided by the RanGTPase system, where cargo is released within the nucleus upon RanGTP binding to Kapβ [13]. Conversely, the binding of export cargo is aided by the binding of RanGTP, where the subsequent release of cargo into the cytoplasm is triggered by RanGTP hydrolysis.

Not all Kapβs recognize their cargo directly. For example, Importin-β1 (Imp-β1) mainly recognizes its cargo through the adapter Importin-α (Imp-α). This pathway is commonly referred to as the classical nuclear import pathway, and involves recognition of a cargo’s NLS by one of the seven human Imp-α isoforms, which are then shuttled through the NPC by Imp-β1 as a heterotrimeric complex [14,15]. NLSs recognized by Imp-α are referred to as classical NLSs (cNLS), are rich in basic amino acids, and come in two forms, monopartite or bipartite [16]. Monopartite cNLSs are comprised of a single cluster of basic amino acids, while bipartite cNLS have two clusters of basic amino acids separated by a linker region of variable length and composition. These motifs can be exemplified by the SV40 Large-T antigen (PKKKRKV) and nucleoplasmin (KRPAATKKAGQAKKKK) cNLS, respectively [17,18]. Structurally, Imp-α is composed of 10 armadillo (ARM) domains which form two pockets, referred to as the major and minor groove [19,20]. These grooves accommodate the basic clusters of amino acids characteristic of a cNLS. Monopartite motifs primarily bind the major groove. However, several NLSs, both cellular and viral, can bind the minor groove exclusively [21,22,23,24]. Bipartite cNLSs occupy both the major and minor grooves, with the smaller N-terminal basic cluster bound to the minor groove and the larger basic cluster bound to the major groove [20].

Another class of NLS that has been characterized is the PY-NLS, which is recognized by transportin-1 and -2 (TNPO1 and TNPO2), both importin Kapβ members [11,25]. The PY-NLS is not as well characterized as the cNLS; however, some general rules have emerged. These include an N-terminal motif, either hydrophobic or basic, and a C-terminal R/K/H-X_2-5_-PY motif, and are found within a structurally disordered region with overall basic charge [25]. An additional Kapβ, transportin-SR2 (also known as TRN-SR or TNPO3), has been shown to import SR splicing factors by binding their arginine-serine (RS) domain [26]. With regard to exportin Kapβs, only one class of NES has been characterized, and this is mediated by exportin-1 (XPO1, also known as CRM1), which recognizes 10–15 amino acid long leucine rich motifs [27,28,29]. Similar to the classical nuclear import pathway, XPO1/CRM1-mediated export has been extensively studied with hundreds of characterized cargo from human and model organisms [30].

The classical nuclear import pathway is assumed to handle the majority of nuclear import as it is the best characterized and has many documented cargoes and cNLSs [16,31]. Sequence attributes of cNLSs have made them highly predictable, and this has led to the development of numerous NLS prediction programs [23,31,32,33,34]. Interestingly, early estimates using PSORT II demonstrated that only ~55% of nuclear proteins in *S. cerevisiae* have a predictable cNLS [16]. As more NLS prediction programs emerged, the fraction of yeast and human nuclear proteins with predictable cNLSs unexpectedly remained between 30% and 40% [31,35]. These observations likely reflect a combination of non-classical import pathways, alternative cNLSs, and piggybacking into the nucleus indirectly via physical interaction with other proteins that directly bind the nuclear transport apparatus. In fact, data from yeast show that up to 50% of proteins that bind Srp1 (the only yeast Imp-α) do not have a predictable cNLS, providing strong circumstantial evidence that their association with Srp1 and subsequent nuclear import occurs via piggybacking or alternative cNLSs [16]. 

Whether or not these observations hold true in humans has not been explored. With the development of databases such as the Human Protein Atlas (HPA) and Human Reference Interactome (HuRI), as well as the abundance of publicly available high-throughput mass spectrometry data, it may be possible to establish a more accurate picture of nuclear transport mechanisms [36,37,38,39]. While examples of piggybacking into the nucleus using Imp-α have been documented for several distinct nuclear proteins, widespread identification of potential piggybacking proteins, or estimates of the extent to which this nuclear import strategy is used, remain poorly characterized [40,41,42,43,44,45]. Using a collection of resources and NLS prediction programs, we aimed to acquire information regarding the prevalence of piggybacking and the use of alternative nuclear import pathways in eukaryotic cells. Our analyses show that nearly 50% of nuclear proteins in the human proteome do not have a predictable cNLS. We identify a large cohort of proteins found in both the nucleus and cytoplasm which have a predicted NES, but not a predicted cNLS. Examinations of binary interactions for six of the seven known Imp-α isoforms demonstrate that 20–50% of interactors also do not have a predictable cNLS. Furthermore, a reanalysis of publicly available mass spectra files for protein interactions mediated by several Imp-α isoforms showed that up to 50% of cargoes do not have a predictable cNLS. Finally, using this data we specifically focus on several nuclear protein complexes involved in transcription, and show that the majority of proteins belonging to the mediator of RNA polymerase II transcription (Mediator) complex interact with at least one Imp-α, yet do not have a predictable cNLS.

## 2. Materials and Methods

### 2.1. Datasets for Nuclear, Cytoplasmic and Nucleocytoplasmic Proteins

Proteins with experimental evidence of being localized to the nucleus or cytoplasm were downloaded from the Human Protein Atlas (HPA). According to the HPA nuclear localization dataset, this includes the nucleoplasm, nuclear speckles and nuclear bodies, while cytoplasmic localization includes the aggresome, cytosol, cytoplasmic bodies, rods and rings. Proteins present in both the nucleus and cytoplasm were additionally grouped together as nucleocytoplasmic proteins. From here, all canonical protein sequences were retrieved from UniprotKB/Swiss-Prot.

Proteins known to associate with yeast Srp1 were downloaded from BioGrid, while interactors for human Imp-α1, α3, α4, α5, α6 and α7 were retrieved from IntAct [46,47]. Only physical interactions were kept, removing any interactions identified through genetic studies or post-translational modifications. Proteins with binary, or direct, interactions with Imp-α were retrieved from the HuRI database for Imp-α isoforms α1, α3, α4, α5, α6 and α7. All interactors were combined together, and redundant interactors were removed before screening against the HPA nuclear dataset in order to obtain those with evidence of nuclear localization only.

### 2.2. NLS and NES Prediction

For the identifying of cNLSs using regular expression matching, we used the regular expressions provided through the eukaryotic linear motif (ELM) database, corresponding to monopartite core (ELME000270), monopartite core with C-terminal preferences (ELME000278), monopartite core with N-terminal preferences (ELME000271) and bipartite (ELME000276) [48]. Our criteria for having a cNLS only required a protein to have at least one of the following regular expressions satisfied.

For predicting NLSs using NLStradamus, each protein was searched for both monopartite and bipartite NLSs using a threshold score of 0.6. All hits were combined, and duplicate protein ID matches were removed to end up with a list of unique proteins containing either a monopartite or bipartite NLS. For searches using cNLS Mapper, the default cut-off score of 0.5 was used and included searching the entire region of the protein for bipartite NLS with a long linker region. Since NLSdb searches for matches within its own library of potential or experimentally confirmed NLSs, no threshold or cut-off scores could be used a priori, and any matches to NLSdb were taken as a hit. Predictions made with NESmapper used the default threshold score of 2 to identify potential NESs. Similar to NLS prediction, any duplicate protein IDs were removed to obtain a list of unique proteins with at least one predicted NES. For more detailed cNLS analysis PONDR and DisEMBL were used to screen for intrinsic disorder [49,50]. PONDR (VSL2) was used to find short (<30 residues) or long (>30 residues) regions of predicted disorder where a given cNLS was located. Similarly, DisEMBL was used to identify regions with a high degree of mobility that overlap the cNLS in question.

### 2.3. Identification of Novel Motifs with MEME and SLiMSearch

Imp-α interactors from the HuRI dataset without a cNLS were combined into a single group and all duplicate protein identifications were removed as well as any nucleoporins or Imp-α proteins. This produced a group of 10 unique proteins which were then analyzed using the motif elicitation program MEME [51]. MEME settings were set to identify three clusters with zero or one occurrence per sequence, with a minimum length of six amino acids. The top scoring cluster was subsequently used for further analysis. Using the motif defined as KxRxHxK, we searched the human proteome with SLiMSearch [52] for any motif matches located within an intrinsically disordered region (disorder cut-off set to 0.4). Identifications retrieved with SLiMSearch then underwent gene ontology analysis using Metascape [53] to identify enriched cellular process.

### 2.4. Proteomics Analysis

Raw mass spectra were downloaded from the Proteomics Identification database (PRIDE) corresponding to project PXD007976 titled “Landscape of nuclear transport receptor cargo specificity” [54,55]. Specifically, mass spectra corresponding to wild-type control, BirA* control, Imp-α1 (N- and C-terminal BirA* tag), Imp-α5 (N- and C-terminal BirA* tag) and Imp-α6 (C-terminal BirA* tag) were retrieved for samples that were digested on-bead. Tandem mass spectra were searched using MS-GF+ against the human Swiss-Prot entries from UniProtKB (release 03/2020, 20,305 entries) and included common contaminants in addition to BirA and streptavidin. Additionally, a reverse decoy database was used for false discovery rate estimation. MSGF+ search parameters were as follows: full tryptic specificity, precursor mass tolerance of 20 ppm, and dynamic modifications for methionine oxidation, N-terminal lysine acetylation and biotinylation of lysine. 

Proteins were identified using a target–decoy strategy with IDPicker and filtered at a false discovery rate of 1% and a minimum of two unique peptides per protein. Experiments where Imp-α was expressed with BirA* either on the N- or C-terminus were combined to establish a unique set of interactors encompassing both experiments. Proteins identified from each experimental sample were analyzed with SAINTexpress [56] using wild-type and BirA* samples as controls. To further increase the statistical strength of identifying co-purifying bait proteins we used additional controls provided through the CRAPome (CC532) [57]. Peptides identified through SAINTexpress with a false discovery rate less than 5% were considered statistically relevant. Finally, all interactors for Imp-α1, α5 and α6 were combined and reduced into a list of non-redundant proteins that could be screened against the HPA for evidence of nuclear localization, unless otherwise stated. Only those with evidence of nuclear localization were used for analysis. For TAF-Imp-α interactions, spectral counts and false discovery rate calculations produced by SAINTexpress were submitted to ProHits-viz for visualization [58].

## 3. Results

### 3.1. Many Nuclear Localized Proteins Do Not Have a Predictable cNLS

**Figure 1:**
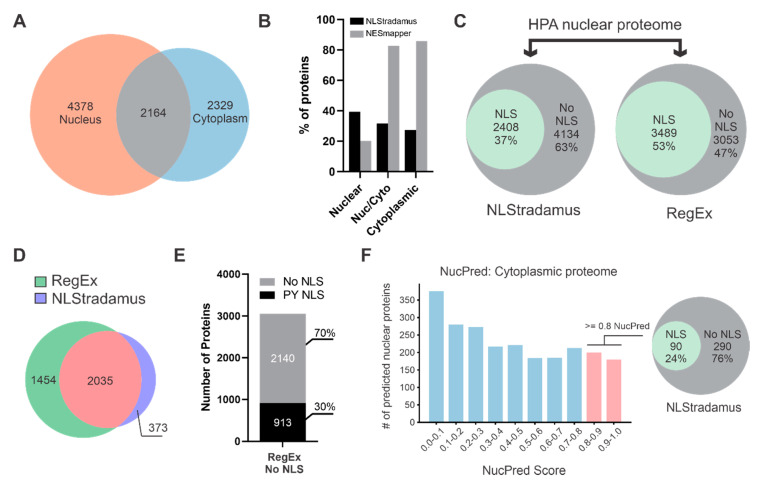
The majority of human nuclear proteins do not contain a predictable cNLS. (**A**) Nuclear and cytoplasmic proteins were retrieved from the Human Protein Atlas (HPA) for the identification of distinct nuclear, cytoplasmic and nucleocytoplasmic proteins. (**B**) cNLS and NES prediction of nuclear, nucleocytoplasmic (Nuc/Cyto) and cytoplasmic proteins from the HPA using NLStradamus and NESmapper, respectively. The majority of Nuc/Cyto and cytoplasmic proteins have a predictable NES in contrast to nuclear and Nuc/Cyto proteins where the majority do not have a predictable cNLS. (**C**) Comparison of cNLS prediction approaches using NLStradamus and regular expression matching (RegEx) on nuclear proteins from the HPA. All motifs corresponding to cNLSs in the eukaryotic linear motif database were used for RegEx matching, where any protein with at least one match is counted as a hit. Comparing approaches shows that somewhere between 47% and 63% of nuclear proteins do not have a predictable cNLS. (**D**) The prediction of proteins with a cNLS using either RegEx matching or NLStradamus demonstrates significant overlap. The majority of NLStradamus predictions are also predicted by RegEx matching. (**E**) Proteins without a cNLS, as determined by RegEx matching, were searched for a minimal PY-NLS (R/H/K-X_2-5_-PY), demonstrating that a substantial portion of nuclear proteins also do not contain a PY-NLS. (**F**) Cytoplasmic proteins were analyzed with NucPred to predict nuclear localization and those with a score greater than 0.8 were searched for cNLSs. Those with a cNLS are considered to have a high probability of nuclear localization, highlighting the potential for additional nucleocytoplasmic localizations not supported by the HPA.

The classical nuclear import pathway is assumed to handle the majority of protein nuclear import. Extensive research into this pathway has established a defined set of rules for the cNLS-Imp-α interaction, making them highly amenable to computational prediction [23,35]. To estimate the fraction of nuclear proteins with a predictable cNLS, we first generated a list of proteins from the Human Protein Atlas (HPA) that are localized to the nucleus. Additionally, we collected proteins that localize to the cytoplasm, in order to capture proteins present in both compartments that could potentially shuttle bidirectionally across the nuclear envelope. From the HPA, 6542 nuclear and 4493 cytoplasmic proteins were identified. These nuclear and cytoplasmic proteins demonstrated substantial overlap, with over 2100 proteins found in both the nucleus and cytoplasm (Figure 1A). This number represents almost a third of the nuclear proteins and one-half of the cytoplasmic proteins that localize to both cellular compartments.

The analysis of proteins that localize to the nucleus, nucleus and cytoplasm, and cytoplasm, using NLStradamus for NLS prediction and NESmapper for NES prediction, indicated that NLSs are more frequently predicted in nuclear proteins and NESs are more frequently predicted in cytoplasmic proteins, as anticipated (Figure 1B). The difference in frequency of identifying a predicted cNLS for nuclear proteins or a predicted NES for cytoplasmic proteins is particularly intriguing. Over 80% of cytoplasmic proteins have a predictable NES, while only ~40% of nuclear proteins have a predictable cNLS.

As less than half of the nuclear proteins have a predicted cNLS, we wondered if this was due to NLS prediction being too specific. To search for cNLSs with greater sensitivity, we used simple regular expression (RegEx) matching to search all nuclear proteins, including those that are nucleocytoplasmic. For RegEx matching, we used experimentally validated motifs corresponding to monopartite core, monopartite N-extended, monopartite C-extended and bipartite from the eukaryotic linear motif database (ELMdb) [48]. As expected, RegEx matching increased cNLS prediction sensitivity; however, putative cNLSs were still identified in only 53% of nuclear proteins, compared to 37% using NLStradamus (Figure 1C). A comparison of proteins with a predicted cNLS from RegEx matching or NLStradamus shows significant overlap, with the majority of NLStradamus hits also being identified by RegEx matching (Figure 1D). Our prediction with NLStradamus agrees with previous findings [31], and less stringent searches for cNLSs using RegEx matching still fail to predict a cNLS in almost 50% of nuclear proteins.

While other NLSs, such as the PY-NLS, exist, only a limited number of PY-NLSs have been characterized in detail, and no reliable prediction models exist. Most PY-NLSs characterized to date possess the sequence motif R\K\H-X_2-5_-PY, where a positively charged amino acid (Arg, Lys, His) can be found up to 5 amino acids N-terminal to a PY motif [59]. While this motif is one of several PY-NLS attributes, it is not sufficient for predicting a PY-NLS, and on its own would be highly over-predictive. Nevertheless, we used this motif to search proteins that do not contain a cNLS using RegEx matching, and found only 30% of these proteins contained this minimal PY-NLS motif (Figure 1E), leaving a substantial portion of the nuclear proteome without a predictable cNLS or PY-NLS.

Despite the HPA characterizing protein subcellular localization in several cell lines, this does not rule out additional cytoplasmic proteins that could potentially localize to the nucleus under different cellular conditions, or other cell types not captured by the HPA. As previously demonstrated, putative cNLSs can be found in over 20% of cytoplasmic proteins (Figure 1B). With this subset of cytoplasmic proteins, we used NucPred to predict each protein’s probability of localizing to the nucleus. NucPred scores range from 0 to 1, with higher scores having a greater probability of a protein being nuclear. As expected, most cytoplasmic proteins had a lower NucPred score; however, many proteins still scored greater that 0.8 (Figure 1F). As these scores are only probabilities, NucPred performance is further enhanced if a protein also has a predicted NLS. Proteins with a NucPred score greater than 0.8 and a predicted NLS have been shown to be correctly identified as nuclear with over 90% accuracy [33]. Taking the 380 cytoplasmic proteins with scores equal to or greater than 0.8, and filtering with NLStradamus, resulted in nearly 25% of these proteins having a potential cNLS. Indeed, it is possible that many of these proteins have nuclear functions despite being classified as cytoplasmic, based on Protein Atlas data. Nevertheless, these observations suggest that substantially more cytosolic proteins may have undercharacterized, context-specific occupancies within the nucleus than anticipated, which cannot be captured by immunofluorescence alone.

Based on data from the HPA, these findings point to a conservative estimate where almost 50% of nuclear proteins lack a predictable cNLS, and this estimate increased to over 60% using more stringent NLS prediction programs. Furthermore, analysis of cytoplasmic proteins using nuclear localization and NLS prediction demonstrates a substantial portion of cytoplasmic proteins may have currently uncharacterized, potentially context-dependent roles within the nucleus. Taken together, these findings emphasize the discrepancy in cNLS prediction for established human nuclear proteins, and highlight an intriguing inconsistency between the frequencies of NES and NLS prediction.

### 3.2. Many Imp-α Binding Partners Do Not Have a Predictable cNLS

**Figure 2:**
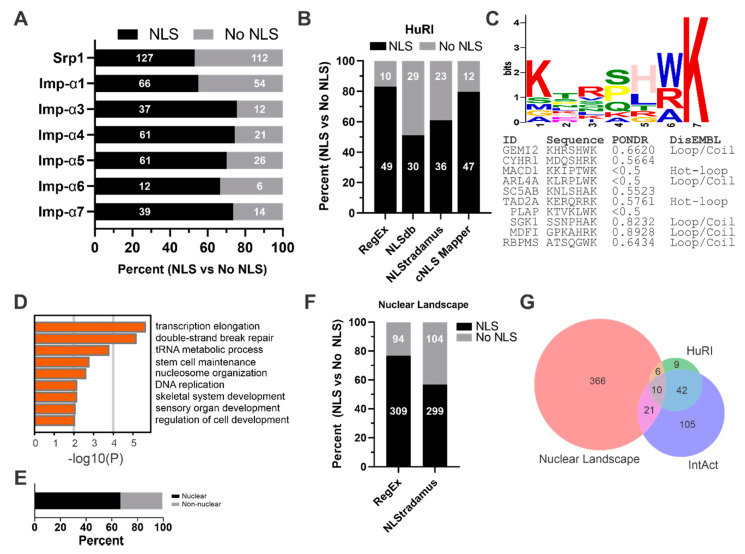
Many Imp-α cargo do not have a predictable cNLS. (**A**) Physical protein interactions, either direct or indirect, for yeast Srp1 and the indicated human Imp-α isoforms were retrieved from BioGrid and IntAct, and analyzed for cNLSs using RegEx matching. Prediction shows between 50% and 80% do not have a predictable cNLS. (**B**) Direct protein interactions for all Imp-α isoforms except Imp-α8 were retrieved from the Human Reference Interactome (HuRI). Interactors were pooled to remove redundant proteins, and checked against the HPA for evidence of nuclear localization before cNLS prediction. Several prediction programs were used to determine a range of predicted cNLSs, which demonstrated between 50% and 80% do not have a cNLS. (**C**) Proteins without a predicted cNLS from any of the prediction programs were processed with MEME to identify novel motifs common amongst each protein that might interact with Imp-α. Several of the motifs identified were rich in basic amino acids, but did not resemble a cNLS. Disorder prediction using PONDR (VSL2) and DisEMBL shows that these motifs are also found within disordered protein regions. (**D**) The motif KxRxHxK was searched against the human proteome using SLiMSearch, identifying 37 proteins, which were then analyzed with Metascape. Proteins bearing this motif are most enriched in core nuclear processes like RNA pol II transcription and DNA repair. (**E**) Proteins with the KxRxHxK motif were also checked against the HPA for evidence of subcellular localization. Of the 30 proteins with localization information, two-thirds have evidence of nuclear localization. (**F**) Reanalysis of the tandem mass spectra for protein interactions corresponding to Imp-α1, α5 and α6 from the Nuclear Landscape dataset. All significant interactions were checked against the HPA for nuclear localization before cNLS prediction. Between 50% and 75% of Imp-α cargoes do not have predictable cNLS when analyzed with NLStradamus and RegEx matching, respectively. (**G**) Identified proteins from the Nuclear Landscape dataset were compared to those from HuRI and IntAct. Comparison shows that the majority of protein identifications from the Nuclear Landscape dataset are not represented within these databases, and that these interactions show similar results in the number of proteins without predictable cNLSs.

Protein nuclear import is mediated by a variety of different importins, ranging from Imp-α and the classical nuclear import pathway to alternative import pathways using importin Kapβs [16,59]. The lack of predictable cNLSs in nuclear proteins may partly be reflected by the diversity of nuclear import pathways; however, previous observations in yeast have shown that up to 50% of proteins which bind Imp-α do not have a predictable cNLS [16]. To evaluate if this holds true for human Imp-α isoforms, we specifically looked at proteins that have documented interactions with an Imp-α family member. To obtain a list of these physical interactors, proteins were retrieved from BioGrid and IntAct databases for the only yeast Imp-α, Srp1, and all seven human Imp-α isoforms [46,47]. To determine which cargoes have a predictable cNLS, we used the less stringent RegEx matching to come up with a conservative list of proteins that interact with Imp-α, but do not have a predicted cNLS (Figure 2A). In yeast, approximately 50% of the proteins which associate with Srp1 have a predictable NLS, and this is in agreement with previous reports [16,35]. Human Imp-α1 shows a similar trend to Srp1, where just over 50% of interactors have a predictable NLS. This is in contrast with Imp-α3, α4, α5, α6 and α7, where roughly 25% of their identified interactions do not have a predictable cNLS. Based on these findings, we conservatively estimate that roughly 25–50% of Imp-a cargo in humans does have a predictable cNLS.

Not all protein interactions reported in databases such as BioGrid or IntAct are binary, making it difficult to determine if a protein is directly binding Imp-α, or if it does so indirectly by piggybacking on a protein that interacts directly with Imp-α. To evaluate direct binding partners of Imp-α, we explored the recently published HuRI database [38]. This project involved a yeast two-hybrid pipeline that tested roughly 17,000 human ORFs in an ‘all-by-all’ format. From this dataset we were able to retrieve 102 non-redundant binary interactions from all Imp-α isoforms, except Imp-α8, which has no data available. Further refinement ultimately reduced this down to 59 proteins, as only 67 show evidence of nuclear localization from the HPA, and further 8 are either nucleoporins or importins. Searching these proteins for potential cNLSs using several approaches revealed that between 20% and 50% do not have a predictable cNLS (Figure 2B). Both RegEx matching and the cNLS Mapper predicted cNLSs in roughly 80% of proteins, while NLSdb and NLStradamus predicted 50–60% with a cNLS, likely putting the range of true cNLSs somewhere between the two extremes.

Despite the HuRI dataset being relatively small compared to the number of potential nuclear proteins that may bind Imp-α, these findings demonstrate that a potentially large fraction of Imp-α binary interactions may be mediated by a non-typical cNLS. To explore this idea further, we used the motif elicitation program MEME to determine if this group of proteins from the HuRI dataset has any common motifs [51]. First, proteins without a predictable cNLS from each prediction program were combined and reduced into a group of 10 non-redundant proteins. These proteins were then evaluated using MEME to look for minimal motifs that occur once in each protein. Interestingly, the top scoring motif was still enriched with positively charged amino acids, despite no resemblance to a true cNLS (Figure 2C). This seven-amino acid motif has the strongest preference for Lys at positions 1 and 7, and for His at position 5. Position 6 was consistently either Trp, Arg or Ala, and position 3 has a minor preference for Arg. Since short motifs, like the cNLS, are most frequently found within intrinsically disordered regions of a protein, we next searched each protein using the disorder prediction programs DisEMBL and PONDR [49,50,60]. Results from PONDR (VSL2) show that many of the motifs are within a predicted region of disorder, based on their score being greater than 0.5. Analysis with DisEMBL was similar, with most motifs residing in predicted disordered loops/coils or hot-loops. Overall, this data from a small subset of proteins shows that an alternative motif, divergent from a cNLS yet possessing several basic residues, may be present.

From the motif generated with MEME, we searched the human proteome for KxRxHxK (since these were the prominent basic amino acids) using SLiMSearch [52]. This resulted in 37 proteins in which this motif could be found within an IDR. Gene ontology analysis of these proteins using Metascape [53] shows that they are most enriched for core nuclear processes involving RNA polymerase II transcription and DNA repair (Figure 2D). In total, 30 of the 37 proteins had subcellular localization data from the HPA, with 20 having evidence of nuclear localization (Figure 2E). Taken together, these findings suggest that most proteins bearing the KxRxHxK motif are likely nuclear.

Proteins known to associate with Imp-α that can be collected from databases such as IntAct or BioGrid likely only represent a fraction of Imp-α cargo. To extend these findings further, we explored datasets which were not available, or not utilized, during previous attempts at characterizing the classical nuclear import pathway in this manner [16,31]. To do this, we reanalyzed publicly available raw mass spectra files published by Mackmull et al., which were obtained through the Proteomics Identification Database (PRIDE) and are referred to here as “Nuclear Landscape” [54]. This dataset includes interaction data for Imp-α1, α5 and α6 that were acquired through in situ proximity ligation (BioID). In their experiments, Imp-α1 and α5 were expressed as N- and C-terminal BirA* fusions, while Imp-α6 was only expressed with C-terminal BirA*. This approach is highly sensitive, and allows protein–protein interactions to be mapped under normal cellular conditions. Briefly, raw tandem mass spectra were searched using MS-GF+ with a reverse target–decoy strategy, and the resulting peptides were assembled into proteins using IDPicker, with a global protein FDR < 1% [61,62,63]. Statistically significant interactions were identified using SAINTexpress, with additional background controls provided through the CRAPome, ultimately resulting in a total of 502 high-confidence interactions [56,57]. This list of interactors was then compared to proteins localized to the nucleus according to the HPA, resulting in a final list of 403 interactors. Many of the proteins omitted show evidence of nuclear localization; however, for consistency, only proteins with evidence in the HPA were used. To establish an estimate of cargoes without a predictable cNLS, we used RegEx matching and NLStradamus to determine that roughly 20–25% and 50% of proteins did not have a predicted cNLS, respectively (Figure 2F). These findings echo the results obtained from the HuRI and IntAct datasets (Figure 2A,B), which show a similar number of proteins without a cNLS when using both prediction approaches. It remained possible that these similarities arise due to the analysis of overlapping/redundant proteins within their respective datasets. However, a comparison of Imp-α interactors from each source demonstrated minimal overlap between proteins identified through our reanalysis and IntAct or HuRI (Figure 2G). Thus, reanalysis of the Nuclear Landscape dataset using both a different mass spectrometry pipeline and statistical protein–protein interaction analysis identified significantly more Imp-α cargoes, many of which are novel, yet also do not have a predictable cNLS.

Overall, using several different cNLS prediction programs, we determined that 20–50% of proteins which directly bind Imp-α do not have a predicted cNLS. Importantly, these observations are independently observed in the reanalysis of the Nuclear Landscape dataset, which represents hundreds of new Imp-α cargoes. When taken together, these data highlight potentially new Imp-α binding motifs, and are also highly suggestive that piggybacking strategies are used extensively for Imp-α interactions.

### 3.3. Identification of Putative Piggybacking Proteins

As shown above, roughly 60% of proteins known to localize to the nucleus do not have a predictable cNLS. Some of these proteins without a cNLS may instead target one of the importin Kapβs directly. However, there remain many nuclear proteins that associate with Imp-α, as determined by proteomic studies, which do not have a predictable cNLS. One situation that would satisfy nuclear import via Imp-α, without the use of a cNLS, is the process of piggybacking, which is simply the indirect association with Imp-α via an intermediary protein [42]. Despite a few specific examples of piggybacking as a mechanism of nuclear import, the prevalence of this process remains poorly characterized.

**Figure 3:**
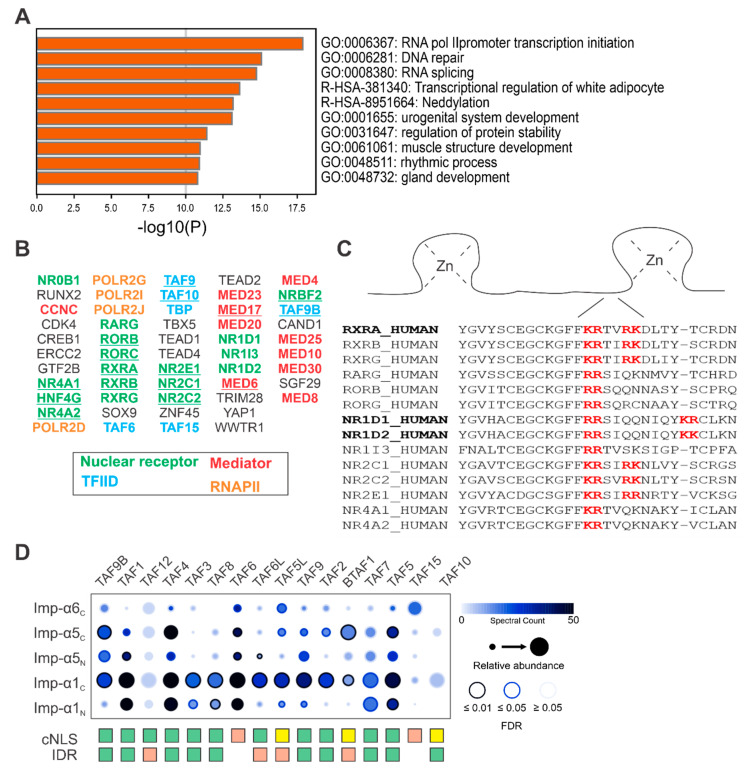
Identification of putative piggybacking proteins in non-cNLS nuclear proteins. (**A**) Nuclear proteins from the HPA without a predicted cNLS were analyzed using Metascape to identify enriched cellular processes. (**B**) Many of the proteins within the RNA polymerase II (RNAPII) transcription initiation cluster belong to related protein groups, such as the nuclear receptors, and distinct multi-protein complexes like transcription factor II D (TFIID), RNAPII and Mediator. Underlined proteins have a predicted cNLS as determined by cNLS Mapper. RNAPII is known to use piggybacking as well as several subunits of TFIID. (**C**) Although not suspected to piggyback, multiple sequence alignment with Clustal Omega of the identified nuclear receptors shows conservation of a motif (red) that has been previously shown to mediate nuclear import (bolded black) [68]. (**D**) Visualization of TAF interactions with Imp-α1 and 5 (N- and C-terminal BirA* fusions) and Imp-α6 (C-terminal BirA* fusion) shows that many TAFs are strongly associated with Imp-α1. Several have a cNLS Mapper score ≥ 7 (green) while others have weaker cNLS Mapper scores that are <7 but still greater than 5 (yellow). Those in red have scores below 5. Importantly, many of these predicted cNLSs are found within disordered regions (green) as determined by PONDR (VSL2).

To identify putative piggybacking proteins and gain an estimate of their relative frequency, we used Metascape to first establish a general overview of the cellular process associated with nuclear proteins from the HPA without a cNLS [53]. The rationale for this being that proteins involved in similar cellular processes are most likely to function together. Of the top 10 non-redundant enriched clusters we identified, the top three processes were RNA polymerase II transcription initiation, DNA repair and RNA splicing, which are all nuclear processes (Figure 3A). Further inspection of members within the RNA polymerase II transcription initiation cluster revealed multiple groups of proteins with related functions (Figure 3B). The first major group consists of proteins belonging to the type-II nuclear receptor family, a class of ligand-regulated transcription factors [64]. Interestingly, most nuclear receptor proteins identified have a cNLS predicted by cNLS Mapper. However, these predicted cNLSs do not align with those identified by experimentation, and are likely incorrect. Interestingly, many nuclear receptors have been shown to contain an NLS within their DNA binding domain, specifically within the linker region between zinc-finger domains [65,66,67]. These motifs appear in many nuclear receptors; however, they do not resemble any previously identified cNLSs or non-cNLSs (Figure 3C).

Additionally, several proteins were identified that function together in large multi-protein complexes, including subunits of RNA polymerase II (RNAPII), transcription factor II D (TFIID) and Mediator complexes. The identification of RNAPII subunits is encouraging, as RNAPII is already suspected to assemble within the cytoplasm prior to nuclear import [42,43,69]. Similar to RNAPII, the assembly of TFIID subunits has been proposed to occur within the cytoplasm, and subsequently enter the nucleus through a piggybacking mechanism. Specifically, the cTAF subcomplex, consisting of TAF2-TAF8-TAF10, has been shown to shuttle into the nucleus via Imp-α1 [40]. Our reanalysis of the Nuclear Landscape dataset using SAINTexpress supports these observations, showing statistically significant interactions (FDR < 0.05) between Imp-α1 and several TAF proteins, including TAF2 and TAF8 (Figure 3D). Visualization of these interactions using ProHits-viz shows the highest number of spectral counts between Imp-α1 (C-terminal BirA* fusion) and various TAF proteins [58]. The N-terminal BirA* fusion of Imp-α1 produced many similar interactions, but with fewer spectral counts. Likewise, Imp-α5 N- and C-terminal BirA* constructs identified similar hits with varying spectral counts, while Imp-α6 produced the fewest hits overall. Despite the positive identification of peptides corresponding to TAF10, the interaction between Imp-α1 and TAF10 was not statistically significant according to SAINTexpress. However, with prior knowledge of a TAF2-TAF8-TAF10 complex and a number of other interactions between Imp-α1 and several TAFs, the Imp-α1-TAF10 interaction is likely accurate. Additionally, many individual subunits of the 5TAF (TAF4, 5, 6, 9 and 12) and sTAF (TAF1, 7, 11 and 13, and TBP) subcomplexes appear to preferentially associate with Imp-α1. Interestingly, despite ample evidence in support of piggybacking, many TFIID subunits have predictable cNLSs within a predicted intrinsically disordered region. With the exception of TAF15, which has a PY-NLS, only TAF6 has no predictable cNLS [70].

Based on these findings, our analysis of nuclear proteins without a predictable cNLS identified protein subunits of RNAPII and TFIID already shown to piggyback into the nucleus. In contrast, many of the Mediator proteins identified do not have a predictable cNLS, a particular area that has remained largely unexplored and could possibly represent a novel example of piggybacking.

### 3.4. Mediator Proteins Associate with Imp-α and Do Not Have a Predictable cNLS

Mediator, like RNAPII and TFIID, is a multiprotein complex consisting of up to 30 subunits. Despite being relatively well characterized with respect to its role in transcriptional coactivation, the nuclear import of Mediator proteins has not been studied extensively. Furthermore, evidence of cytoplasmic assembly prior to nuclear import via a piggybacking mechanism has not been previously proposed.

**Figure 4:**
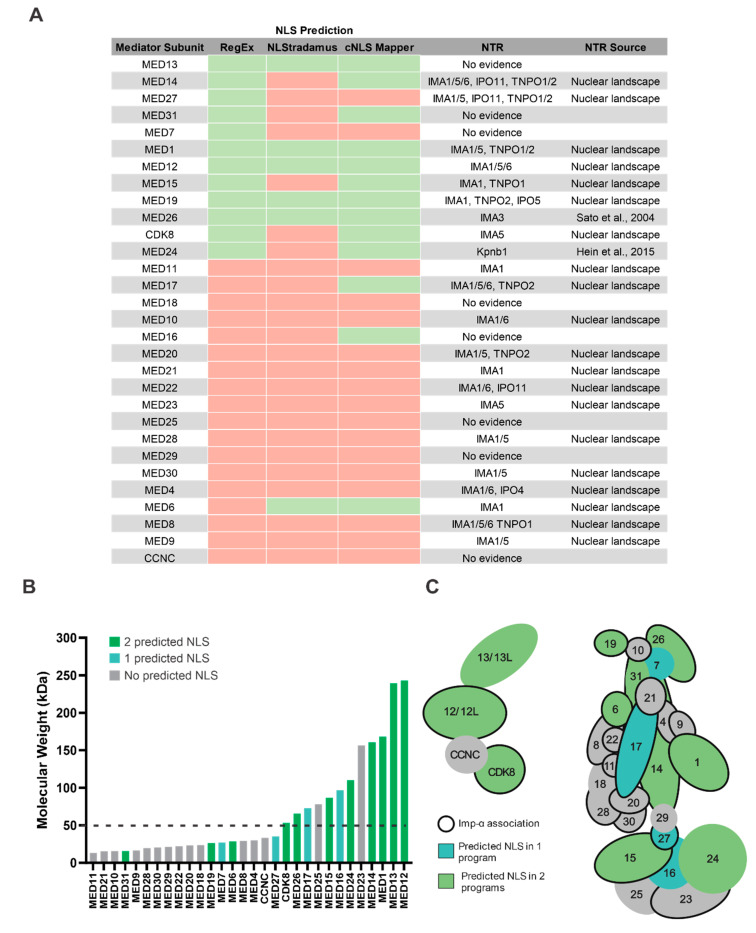
Mediator complex subunits may utilize a piggybacking mechanism for nuclear import. (**A**) Mediator subunits were analyzed for cNLSs using different cNLS prediction programs, and data was tabularized using Microsoft Excel. Many subunits have a predicted cNLS (green) from more than one program, while the majority do not have a predicted cNLS (red). Data from the Nuclear Landscape dataset and other published nuclear transport receptor (NTR) interactions show that many subunits associate with Imp-α, as well as transportin (TNPO). (**B**) Mediator subunits vary in molecular weight, with larger subunits more frequently having a predicted cNLS. Subunits with a cNLS predicted from two programs or more are shaded in dark green (2 NLS) and those with a prediction from only one program are shaded in light blue (1 NLS). Although imprecise, a passive diffusion limit of 50kDa (dotted line) shows that many subunits without a cNLS are below this cut-off. (**C**) A model figure of Mediator was adapted from Soutourina, 2018, to show corresponding subunits with predicted cNLSs as well as Imp-α associations.

To investigate the Mediator complex further, we first inspected each Mediator subunit for a predictable cNLS using RegEx matching, NLStradamus and cNLS Mapper (Figure 4A). Of the 30 Mediator subunits evaluated, RegEx matching was the most sensitive, identifying 12 proteins with a cNLS, most of which were confirmed with NLStradamus and/or cNLS Mapper. Of the remaining 18 Mediator subunits without a RegEx-predicted cNLS, only 3 were predicted to have a cNLS using one of the other prediction programs. Overall, using each cNLS prediction method, only 11 of the 30 proteins have a cNLS predicted by at least two approaches, suggesting many subunits may use alternative nuclear import pathways, alternative cNLSs, or possibly piggyback into the nucleus.

In addition to cNLS prediction, we inspected the Nuclear Landscape dataset along with a literature search for interactions between Mediator proteins and nuclear transport receptors. In addition to Imp-α1, α5 and α6, the Nuclear Landscape dataset also contains information for other nuclear transport receptors, and includes several importin Kapβ proteins (Kpnb1, IPO4, IPO5, IPO11 and IPO13) as well exportin Kapβs (NXT1, NXT2, XPO1, XPO2 and XPO7). From this dataset, 22 components of the Mediator complex were identified as having an association with at least one nuclear transport receptor (Figure 4A). Although most Mediator subunits interact with at least one Imp-α protein, many do not have anything resembling a cNLS. For MED7 and MED27 specifically, putative cNLSs were identified using RegEx, but not NLStradamus and cNLS Mapper, suggesting these cNLSs may not be valid. Interestingly, while many Mediator proteins associate with multiple Imp-α isoforms, or importin Kapβ transporters like TNPO1 and 2, and IPO 4, 5 and 11, none exclusively associate with only Kapβ proteins. In other words, these Mediator associations always co-occur with an Imp-α.

Due to the physical limitations imposed by the NPC, the nuclear import of larger proteins requires facilitated nuclear transport pathways. Individual Mediator components range from 13kDa to over 200kDa. Not surprisingly, as molecular weight increases, so does the likelihood of a protein having a predictable cNLS (Figure 4A,B). Most Mediator subunits without a predictable cNLS are less than 50kDa, and in theory may enter the nucleus via passive diffusion. In contrast, both MED23 and MED25 exceed the NPC diffusion limit and lack a predicted cNLS. Given the extensive number of interactions made within the Mediator complex (Figure 4C), it is plausible that MED23, MED25 and many of the smaller components lacking cNLSs piggyback into the nucleus with the larger cNLS-bearing subunits.

Based on these analyses, it appears that the classical nuclear import pathway is responsible for the nuclear import of the majority of Mediator subunits, while alternative pathways using Kapβs may be used to a lesser extent. It is particularly interesting that most Mediator subunits associate with the classical nuclear transport receptor Imp-α, yet do not have anything resembling a cNLS, suggesting that Mediator components may piggyback into the nucleus as complexes, as described for RNAPII and TFIID.

## 4. Discussion

Here, we perform a general analysis of protein nuclear import, and highlight several novel and interesting observations. In general, our results extend previous findings found in model organisms to provide evidence that nuclear import signals are absent in a major fraction of the human nuclear proteome.

Overall, our approach used RegEx matching to identify predicted cNLSs within the human nuclear proteome, which demonstrated that approximately 50% of nuclear proteins from the HPA have a predictable cNLS. Importantly, these findings are based on the assumption that each predicted cNLS is accessible to Imp-α, and resides within an IDR. Indeed, many predicted cNLSs likely reside within an IDR and are non-functional; however, the primary objective was to identify proteins without a cNLS. Applying IDR prediction to proteins without a predictable cNLS would not provide any additional information, and therefore, these assumptions were necessary for creating a high-confidence, conservative list of non-cNLS bearing nuclear proteins. Based on these findings, NLS prediction using RegEx matching and NLStradamus suggests that somewhere between 47% and 63% of nuclear proteins from the HPA do not have a predictable cNLS.

Analysis of nuclear proteins obtained through the HPA showed a large discrepancy between the presence of predicted cNLSs (<40%) in nuclear proteins and predicted NESs (>80%) in cytoplasmic proteins. It is unlikely that cNLS prediction is simply worse than NES prediction, given the fact that both types of motifs have been extensively studied. Rather, this could reflect the diversity in pathways that control protein import or export. All proteins are translated within the cytoplasm, and therefore nuclear proteins require a process to reliably pass through the NPC, in contrast to cytoplasmic proteins that function in the same subcellular compartment they are translated in. Interestingly, over 80% of cytoplasmic proteins have a predictable NES, when in theory this is unnecessary. Possibly, many NESs serve to simply export cytoplasmic proteins that may drift into the nucleus, or that become localized to the nucleus upon nuclear envelope reformation after mitosis. In these instances, it is possible that the XPO1 pathway is responsible for dealing with these scenarios. In fact, this line of reasoning is supported by experimental evidence suggesting XPO1-mediated export functions as a countermeasure to help define the nuclear and cytoplasmic compartments [30].

In contrast to NESs, less than 40% of nuclear proteins have a predicted cNLS. NLStradamus uses a relatively stringent statistical model to predict NLSs, and this includes both cNLSs and non-cNLSs that could bind Kapβs. For this reason, we also used a non-statistical approach that uses simple regular expression matching (RegEx), which likely over-predicts many cNLSs. Paradoxically, the over-predictive nature of this approach is well suited for finding proteins without anything resembling a cNLS. Surprisingly, RegEx matching only identified cNLSs in 53% of nuclear proteins, whereas NLStradamus predicted cNLSs in only 38% of proteins, which is similar to results obtained from the analysis of 2163 human nuclear proteins with NLSdb [31]. Based on these findings, we conservatively estimate that at least 50% of nuclear proteins in humans do not have a cNLS. Roughly one-third of these proteins are predicted to meet one of the requirements of a PY-NLS by having a R/H/K-X_2-5_-PY motif. However, this is only one of the criteria of a PY-NLS, and the large majority of these are probably not true PY-NLSs [11]. Nevertheless, even if these were true PY-NLSs, this leaves a substantial portion of the nuclear proteome without any predictable NLS. Other variants of the PY-NLS exist that do not have the PY motif, or instead have PL in place of PY; however, only a few examples of these exist, and there is no way to determine how abundant these motifs are within the nuclear proteome [45,71,72].

The discrepancy between cNLS and NES prediction is also apparent in nucleocytoplasmic proteins. Hypothetically, these proteins should possess both targeting motifs; however, roughly only 30% contain a cNLS, while ~80% have an NES [73]. Interestingly, this leaves more than 10% of nucleocytoplasmic proteins without either a predictable cNLS or NES. In addition, up to 25% of cytoplasmic proteins have a predicted nuclear localization, as well as a putative cNLS. Although immunofluorescent imaging is highly informative for protein localization, a single image—or even several—only provides information for that particular time and context. It is possible that at least some proteins documented as cytoplasmic have short tenures within the nucleus, in response to a particular stress or stimulus that is not captured through tissue culture-based experiments.

To date, cNLS prediction using human Imp-α binding partners has not been performed. In yeast, it has been shown that 50% of Srp1-binding partners do not have a cNLS [16]. However, because these data were collected using yeast proteins in a yeast system, indirect binding to Srp1 cannot be ruled out. Analysis of Imp-α interactors from BioGrid and IntAct shows that between 50% and 70% do not have a predictable cNLS; however, whether or not these interactions are direct is also unclear. Imp-α data taken from the HuRI database is less likely to be impacted by indirect binding, since binary interactions of human proteins were tested in yeast, and it is less likely for yeast proteins to facilitate human protein interactions. Using either RegEx matching or cNLS Mapper, we determined that at least ~20% of human Imp-α interactors do not have a predictable cNLS. This raises the possibility that a novel, as yet unidentified binding motif is responsible for a subset of Imp-α interactions.

A widely used computational approach for identifying novel motifs is based upon the assumption that multiple unrelated proteins that interact with the same protein are likely to use the same, or a highly similar, interaction motif [74,75,76]. Using proteins from the HuRI dataset without a putative cNLS, we attempted to find a consensus motif using MEME [51]. This identified a motif that was rich in positively charged amino acids. This motif does not conform to a typical cNLS, but the two do share important properties, such as basic amino acids and a localization within predicted disordered protein regions. Using this consensus, we searched the human proteome for the motif KxRxHxK. Interestingly, proteins with this motif are enriched in nuclear processes and show evidence of nuclear localization. The consensus motif identified should be taken with careful consideration, since many of the positions do not have a clear amino acid preference. Additionally, the sequences identified in Figure 2C may be reminiscent of importin-α C-terminal-binding segment (iCBS)-NLSs, which bind a C-terminal region of Imp-α instead of the major or minor grooves, and are rich in basic amino acids, but do not appear to conform to any regular pattern [77].

Data available through resources such as IntAct and HuRI only provide a limited number of Imp-α interactions. For example, the HuRI dataset tested each Imp-α isoform against 17,000 human ORFs, yet reported only ~250 interactions for all Imp-α isoforms combined. These numbers are surprisingly low, considering thousands of proteins are localized to the nucleus. This prompted us to search for additional Imp-α interactions by reanalyzing proteomic data from mass spectrometry repositories, such as PRIDE. Here, we identified a dataset (referred to as Nuclear Landscape) that used BioID, a proximity ligation technique designed to capture protein interactions in vivo, with Imp-α1, α5 and α6 [54,78]. In these experiments, Imp-α was expressed as a fusion protein with BirA* on either the N- or C-terminus. In the presence of exogenously supplied biotin, Imp-α-BirA* will biotinylate proximal proteins in vivo, which can then be subsequently identified through streptavidin-based affinity purification and mass spectrometry. Since proximal and directly interacting proteins are biotinylated directly, this approach is more sensitive in detecting piggybacking interactions than standard affinity purification approaches, as stable interactions are not required during sample preparation. The majority of Imp-α associated proteins identified through this reanalysis are not represented in the HuRI or IntAct datasets. Importantly, RegEx matching shows at least 20–25% do not have a predictable cNLS, in agreement with cNLS prediction performed on proteins retrieved from HuRI and IntAct. Thus, this independent, experimentally based method of detecting Imp-α-associated proteins confirms that many nuclear proteins do not have a predicted cNLS. This may indicate piggybacking into the nucleus, since these interactions would not necessarily be detected through binary interaction studies performed in yeast.

Having established that many nuclear proteins and Imp-α cargo do not have a predictable cNLS, we next wanted to identify putative piggybacking proteins. A Metascape analysis of the cellular processes enriched with nuclear proteins without a predictable cNLS identified RNA polymerase II transcription initiation. Within this group were many proteins belonging to a subfamily of the nuclear receptors. Although not suspected of piggybacking, the alignment of the region located between zinc-fingers shows conservation of an experimentally validated NLS. This NLS has been shown to be active in other nuclear receptors, like the vitamin D receptor, RXR and NR1D1/2 (Rev-Erbα/β) [65,66,67]. The non-classical appearance of this motif, and divergence from other NLSs in general, makes it interesting from a nuclear import perspective, and warrants further investigation.

Intriguingly, several proteins represented within the group of cargo without predicted cNLSs are already known to use piggybacking, and these mainly belong to the RNAPII complex, where assembly has been shown to take place within the cytoplasm prior to nuclear import [43,69]. Additionally, several TAF proteins belonging to the TFIID complex were identified in this group. TAF8 and TAF10 assemble co-translationally within the cytoplasm and shuttle into the nucleus along with TAF2 [40,79,80]. Similarly, TAF6 and TAF9, as well as TAF1 and TBP, assemble co-translationally and may also piggyback into the nucleus [79,81,82]. Analysis of TAF proteins identified in our reanalysis shows that most subunits have a cNLS with a cNLS Mapper score greater than 7, which is considered sufficient to localize GFP to the nucleus. This is an interesting observation considering that many of these proteins are suspected to piggyback into the nucleus as subunits of larger multi-protein complexes. Whether or not these cNLSs are functional, or even accessible to Imp-α, is unknown. However, based on our reanalysis of the Nuclear Landscape dataset, the majority of these subunits associate with Imp-α, suggesting some of these cNLSs may be accessible for binding. It is possible that pre-assembled TFIID is imported into the nucleus in a manner whereby multiple pre-assembled subunits are able to independently contact Imp-α.

In contrast to RNAPII and TFIID, components of the Mediator complex have not been reported to piggyback into the nucleus. Mediator is an evolutionarily conserved multi-subunit complex composed of up to 30 subunits, and it is a key component of transcription regulation [83,84]. Mediator’s main function is to bridge interactions with transcription factors at enhancer regions to the transcriptional machinery assembled at promoters as the pre-initiation complex (PIC) [85]. The composition of Mediator can be subdivided into the head (MED6/8/11/17/18/20/22/28/30), middle (MED1/4/7/9/10/19/21/26/31), tail (MED15/16/23/24/25/27/29) and kinase module (MED12/13, CCNC and CDK8 or CDK19). Intriguingly, the large majority of Mediator proteins do not have a predictable cNLS, but were still observed to associated with Imp-α according to our analysis of the Nuclear Landscape dataset. Based on these findings, it is highly likely that Mediator subunits utilize a piggybacking mechanism similar to RNAPII and TFIID. Furthermore, there appears to be a trend across all Mediator modules wherein smaller subunits may piggyback on their larger cNLS-bearing binding partners. Although these smaller subunits could diffuse into the nucleus, active transport via piggybacking may preserve the stoichiometric ratios and import rates necessary for this essential function. Furthermore, associations between the smaller Mediator subunits and Imp-α clearly support active transport, and not passive diffusion. Of the individual modules, the head module may represent a good starting point for exploring piggybacking, as it had the fewest subunits with a predicted cNLS. MED14, which links the head, middle and tail modules, contains a cNLS, and could possibly nucleate the piggybacking of several Mediator proteins as well [86].

The fact that RNAPII, TFIID and potentially Mediator use piggybacking for nuclear localization is interesting, given that they all function in the formation of the PIC. The assembly of such multi-subunit complexes in the cytoplasm, and the subsequent co-transport via piggybacking into the nucleus, suggests that this may be important for their respective functions. Transport through the NPC is rapid; however, proteins of different sizes transport at different rates [87]. Pre-assembled complexes can traffic at a uniform rate and arrive at the nucleus in a functional format, rather than importing individually at different rates with subsequent piece-by-piece assembly at an enhancer or promoter.

Overall, this data highlights several interesting observations regarding nuclear transport. Although we identified more nuclear proteins with a predicted cNLS than previously reported, at least ~50% of human nuclear proteins do not have a predictable cNLS. We also show for the first time that at least 20% of the proteins that bind a variety of human Imp-α isoforms do not have a predictable cNLS. Taken together, many nuclear proteins likely localize by extensive use of non-classical nuclear import pathways, as well as piggybacking mechanisms. The analysis of nuclear proteins without cNLSs provides additional evidence for the piggybacking of the TFIID complex into the nucleus, and suggests that the Mediator complex similarly piggybacks into the nucleus. Overall, these results demonstrate the need for deeper investigation into alternative NLSs and nuclear piggybacking mechanisms.
